# The Association of Genotype-Based Inbreeding Coefficient with a Range of Physical and Psychological Human Traits

**DOI:** 10.1371/journal.pone.0103102

**Published:** 2014-07-25

**Authors:** Karin J. H. Verweij, Abdel Abdellaoui, Juha Veijola, Sylvain Sebert, Markku Koiranen, Matthew C. Keller, Marjo-Riitta Järvelin, Brendan P. Zietsch

**Affiliations:** 1 School of Psychology, University of Queensland, Brisbane, Queensland, Australia; 2 Department of Biological Psychology, VU University Amsterdam, Amsterdam, The Netherlands; 3 Department of Psychiatry, University of Oulu and Oulu University Hospital, Oulu, Finland; 4 Institute of Health Sciences, University of Oulu, Oulu, Finland; 5 Biocenter Oulu, University of Oulu, Finland; 6 Department of Psychology and Neuroscience, University of Colorado, Boulder, Colorado, United States of America; 7 Institute for Behavioral Genetics, University of Colorado, Boulder, Colorado, United States of America; 8 Department of Epidemiology and Biostatistics, MRC Health Protection Agency (HPA) Centre for Environment and Health, School of Public Health, Imperial College London, United Kingdom; 9 Unit of Primary Care, Oulu University Hospital, Oulu, Finland; 10 Department of Children and Young People and Families, National Institute for Health and Welfare, Oulu, Finland; 11 Genetic Epidemiology, QIMR Berghofer Medical Research Institute, Brisbane, Queensland, Australia; University of Texas School of Public Health, United States of America

## Abstract

Across animal species, offspring of closely related mates exhibit lower fitness, a phenomenon called inbreeding depression. Inbreeding depression in humans is less well understood because mating between close relatives is generally rare and stigmatised, confounding investigation of its effect on fitness-relevant traits. Recently, the availability of high-density genotype data has enabled quantification of variation in distant inbreeding in ‘outbred’ human populations, but the low variance of inbreeding detected from genetic data in most outbred populations means large samples are required to test effects, and only a few traits have yet been studied. However, it is likely that isolated populations, or those with a small effective population size, have higher variation in inbreeding and therefore require smaller sample sizes to detect inbreeding effects. With a small effective population size and low immigration, Northern Finland is such a population. We make use of a sample of ∼5,500 ‘unrelated’ individuals in the Northern Finnish Birth Cohort 1966 with known genotypes and measured phenotypes across a range of fitness-relevant physical and psychological traits, including birth length and adult height, body mass index (BMI), waist-to-hip ratio, blood pressure, heart rate, grip strength, educational attainment, income, marital status, handedness, health, and schizotypal features. We find significant associations in the predicted direction between individuals' inbreeding coefficient (measured by proportion of the genome in runs of homozygosity) and eight of the 18 traits investigated, significantly more than the one or two expected by chance. These results are consistent with inbreeding depression effects on a range of human traits, but further research is needed to replicate and test alternative explanations for these effects.

## Introduction

Inbreeding is mating between related individuals, whereby offspring inherit two copies of the same ancestral gene (autozygosity). Because all members of a species are related to some degree, all individuals are likewise “inbred” to some degree, but there is variation between individuals in how closely or distantly related their parents are. It is widely observed across species that offspring of closely related mates tend to have lower fitness and fitness-related characters [1]. This effect, called “inbreeding depression”, is thought to be primarily due to directional dominance (i.e. deleterious alleles tending to be (partially) recessive); the other possibility, overdominance (i.e. the heterozygote is more fit than either homozygote), is thought to play a more limited role [2].

The theoretical relation of directional dominance to inbreeding depression is that purifying selection is less efficient at eliminating (partially) recessive deleterious alleles than additive or dominant deleterious alleles, so that extant deleterious alleles tend to be more recessive than would be expected due to chance (i.e. directional dominance). When genetically related individuals mate, offspring are at an increased risk of inheriting two copies of recessive deleterious alleles, which would expose the offspring to the full (normally hidden) deleterious effects of those alleles, hence decreasing the fitness of the offspring.

Until recently, inbreeding effects could only be tested using inbreeding (*F*) coefficients based on knowledge of pedigree. In many animals this methodology is feasible and many inbreeding effects have been demonstrated [3], but in many human populations mating between known relatives is rare, making it difficult to obtain large enough samples for a sufficiently powerful test. Nevertheless, evidence of deleterious effects of close inbreeding has been found on traits and diseases including intelligence [4], [5], schizophrenia [6], bipolar disorder [7], hypertension [8], heart disease [9], and cancer [10]. However, there is often social stigma associated with close inbreeding, making it likely that those who mate with a known relative are not a representative sample of the broader population (meaning inbreeding effects could be due to non-genetic confounders). As such, understanding of inbreeding effects in humans from these studies is limited.

Recently, the availability of high-density genotype information has enabled the estimation of inbreeding without any knowledge of pedigree, by looking at the proportion of the genome that occurs in stretches of homozygous DNA (runs of homozygosity; ROHs). Whereas any two individual alleles might be homozygous by chance (“identical by state”), long ROHs are likely to represent two segments that are “autozygous”, or identical by descent from a common ancestor. As such, the proportion of the genome in ROHs (*F*_roh_) can be used to measure autozygosity and thus inbreeding [11]. Earlier studies using relatively few genetic markers have been criticised for not capturing genome-wide autozygosity [12], but current technology enables genotyping of hundreds of thousands of single nucleotide polymorphisms (SNP) across the genome, allowing much more reliable characterisation of autozygosity. Keller et al. [11] detail various advantages of inbreeding coefficients estimated from dense SNP data over those estimated from pedigree, but most importantly the former incorporates autozygosity arising even from very distant common ancestors, which enables testing for inbreeding effects in outbred human populations. However, because of the low variance in inbreeding coefficients in outbred populations, very large samples (e.g., 10K-60K depending on the strength of the effect and the variation in inbreeding) are normally required for sufficient power to detect expected effect sizes [11]. Because of this requirement, only a few traits to date have been associated with inbreeding measured using ROHs in high-density genotype data, including socially less-desirable personality traits (N≈10,000 [13]), schizophrenia (N≈22,000 [14]) and shorter height (N≈35,000 [15]). However, major depressive disorder was not associated with ROHs [16]. Intelligence and educational attainment (which are highly correlated) have been studied in a British and a Dutch sample, respectively, both of N ∼2000; each sample yielded significant associations with ROHs but in opposite directions [17], [18].

Here we aim to gain greater insight into inbreeding depression in humans by testing in one sample the association of ROHs with a range of physical and psychological traits that are potentially related to fitness, so that effects can be gauged across traits without the problem of different traits having been tested in different populations. To this end, we take advantage of a densely genotyped sample (N≈5500) from a relatively isolated population (Northern Finland Birth Cohort 1966 [19]), in which greater variance in the inbreeding coefficient (e.g. the standard deviation of Froh in this sample is ∼3.5 times that of a comparable Australian sample [13]) affords greater than usual power for a sample of this size. We test the association of ROHs with 18 fitness-relevant traits: birth length, adult height, body mass index (BMI), waist-to-hip ratio, blood pressure variables, heart rate, grip strength, educational attainment, income, marital status, handedness, health measures, and potential schizotypal features. Based on the aforementioned evolutionary genetic theory, we expect associations such that individuals with higher inbreeding coefficients have trait values reflecting lower physical and mental health, lower attractiveness (e.g. shorter height in males, higher waist-to-hip ratio in females, unmarried, lower educational attainment and income), and greater developmental instability (e.g. non-righthandedness [20]).

## Methods

### Participants

The Northern Finland 1966 Birth Cohort (NFBC) is a longitudinal population-based birth cohort including 12,058 individuals born in 1966 in the two northernmost provinces of Finland (Lapland and Oulu), which comprised 96.3% of all births [21], see http://www.oulu.fi/nfbc/. For the present study we used data from a postal questionnaire (N = 8767) and a clinical examination (N = 6033) from the 31-year follow-up study obtained in 1997. At the time of the assessment the participants were between 29 and 33 years old (M = 31.3±0.4); this narrow age range obviated the need to control for age in our analyses. The cleaned genotypic dataset consisted of 5368 individuals, 2574 males and 2794 females, but sample sizes differ per variable. Informed consent for the use of the data and DNA was obtained from all subjects, and ethical approval was granted by the Ethics Committee of the Northern Ostrobothnia Hospital District in Oulu (Finland).

### Phenotypic measures

Various physical and psychological measures were obtained from the participants, some by means of questionnaire and others during a clinical examination. Continuous variables were winsorised at three standard deviations from the mean and standardised separately by sex to remove the effect of potential gender differences. Detailed information about the measures and the data cleaning steps per measure can be found in the Supplementary Methods in File S1.

#### Physical and physiological measures

During a clinical examination individual's anthropometrical data, blood pressure, heart rate, and various physical fitness measures were obtained. For this study we used individual's measures on height, BMI, diastolic and systolic blood pressure, heart rate, waist-to-hip ratio, and grip strength.

#### Postal questionnaire data

Participants filled out a mailed questionnaire with questions about their background, about physical exercise and performance capacity, occupation and working history, environment, health, gynaecology, the use of public health services and living habits. For the present study we used self-report data on marital status, household income, educational attainment, birth length, handedness, life satisfaction, self-rated health, lifetime health problems verified by a doctor, and schizotypal features (Physical Anhedonia Scale, Social Anhedonia Scale, and Perceptual Aberration Scale) [22], [23].

### Genotyping; quality control and pruning

DNA samples were collected in accordance with standard protocols and were genotyped on the Illumina 370 duo Chip [24]. The genotype data underwent standard quality control (QC) procedures, including checks for gender mismatch, very low heterozygosity rates (N = 2), unintended 1^st^ or 2^nd^ degree relatedness (Pi-Hat ≥ 0.20), and individual missingness (call rate<95%), resulting in the removal of 178 individuals.

Furthermore, we removed SNPs with a minor allele frequency (MAF) <0.05, with a Hardy–Weinberg equilibrium (HWE) test P<0.001, and a call rate <95% (i.e. missing genotype calls >5%). We then pruned the SNP data lightly (i.e., removing SNPs with a variance inflation factor [VIF] *>*10 using PLINK [25], as recommended by Howrigan et al. [26]). Our final sample included 5,368 individuals and 184,909 SNPs. Note that the sample size differs per variable due to different numbers of missing data across phenotypes. For details of the genotypic data cleaning steps see Table S1 in File S1.

### Measuring inbreeding: Runs of homozygosity

Based on the SNP data for each individual we obtained an index of the level of inbreeding in the individual's ancestry. Runs of homozygosity (ROHs) are homozygous stretches of DNA that can be observed in the offspring of even distant relatives [11], [26]. The ROH calling algorithm (as implemented in PLINK [25]) slides a moving window of a specified number of SNPs across the genome to detect long runs of homozygous genotypes. As such, using the PLINK software, we quantified individuals' level of inbreeding (*F*_roh_) by summing the total length of their genome that is in autosomal ROHs and divide that by the total SNP-mappable autosomal genome length (2.77 × 10^9^).

In this study we defined ROHs (based on recommendations from Howrigan et al. [26], as stretches of at least 65 continuously homozygous SNPs (not allowing any heterozygotes), using lightly pruned SNP data. To minimize underestimation of the number of runs, three (approximately 5%) missing genotypes within an otherwise unbroken homozygous segment were allowed in a run. Further details of the parameters we used for the ROHs analysis can be found in Table S2 in File S1.

### Testing the association between runs of homozygosity and the phenotypic measures

Subsequently, we determined the correlation of inbreeding (*F*_roh_) with each of the phenotypes described above. Sex differences in the phenotypic measures were controlled for in all analyses. We present results controlling for zero, 1, 5, and 10 ancestry-informative principal components (PCs) as obtained from GCTA [27]. We do so because not controlling for ancestry-informative PCs entails the risk of confounding by population stratification (i.e. different ancestral groups might have different levels of inbreeding and different levels of a given trait for reasons not related to the effect of inbreeding on the trait), while controlling for a large number of ancestry-informative PCs entails the risk of removing true inbreeding effects on the traits. We therefore present results with ancestry controls of varying stringency so that the reader has all the information to make their own interpretation of the data.

It is likely that there have been different selection pressures on males and females for height [28], grip strength [29], and waist-to-hip-ratio [30]; for these variables we performed separate analyses by sex in addition to the main analyses with the sexes pooled.

## Results

### Descriptive statistics

Descriptive statistics of all phenotypic measures can be found in the Supplementary Material: Descriptive Statistics, in File S1. Table 1 shows the descriptives of the number of ROHs, and *F*_roh_. Note that the levels of inbreeding are relatively high because the sample is from Northern Finland which has a small effective population size due to historical population bottlenecks [19].

**Table 1:** Descriptive statistics for inbreeding coefficients (number of runs of homozygosity (ROHs) and proportion of genome in runs of homozygosity (*F*_roh_)).

N	Inbreeding measure	Minimum	Maximum	Mean	Median	SD
5368	Number of runs	0	69	14.73	14	5.605
	*F*_roh_	0	.11	.0118	.0101	.0080

### The association between runs of homozygosity and the phenotypic measures

We tested for a correlation between *F*_roh_ and the phenotypic traits. As shown in Table 2, *F*_roh_ correlated significantly (p<.05) and in the expected direction with eight of the 18 phenotypes, including: height, grip strength, household income, educational attainment, birth height, life satisfaction, Physical Anhedonia Scale, and Social Anhedonia Scale. This is significantly more than expected by chance (p = 1.1 *10^-6^). (NB; this probability was obtained with a binomial test in R, in which we calculated the chance of finding 8 significant associations out of 18 traits tested, with an alpha level of .05 (R code: binom.test(8,18,0.05)). For all these traits higher levels of inbreeding are associated with low-fitness trait values. For one scale – lifetime health problems – we found a significant association with inbreeding in the opposite (unexpected) direction: individuals with higher *F*_roh_ score lower on the lifetime health problems scale.

**Table 2:** Correlations between inbreeding coefficients (runs of homozygosity; ROHs) and the various phenotypes.

	N	Expected direction of association	ROHs	ROHs corrected for 1PC	ROHs corrected for 5 PCs	ROHs corrected for 10 PCs
Height	5307	Negative	−.083***	−.068***	−.040**	−.037**
Birth length	5105	Negative	−.042**	−.031*	−.022	−.020
BMI	5289	Positive	.000	−.004	−.009	−.011
Waist-to-hip ratio	5120	Positive	.006	−.004	.005	.001
Diastolic blood pressure	5279	Positive	−.004	−.003	−.004	−.004
Systolic blood pressure	5289	Positive	.010	−.005	.012	.010
Heart rate	5287	Positive	−.016	−.025	−.009	−.009
Grip strength	5195	Negative	−.035*	−.006	−.013	−.011
Handedness^1^	5317	Positive	.016	.013	.013	.016
Marital status^2^	5301	Positive	−.015	−.015	−.024	−.026
Household income	4788	Negative	−.083**	−.063**	−.038**	−.033*
Educational attainment	4609	Negative	−.065***	−.050**	−.038**	−.035*
Life satisfaction^3^	5202	Positive	.027*	.015	.007	.007
Self-rated health^4^	5302	Positive	−.021	−.024	−.018	−.020
Lifetime health problems	5181	Positive	−.029*	−.033*	−.038**	−.038**
Physical Anhedonia Scale	4532	Positive	.046**	.046**	.052***	.050**
Social Anhedonia Scale	4530	Positive	.045**	.044**	.032*	.029*
Perceptual Aberration Scale	4530	Positive	.005	.006	−.012	−.014

Analyses were performed without correcting for ancestry, and with correcting for ancestry by including the first, the first 5, and the first 10 ancestry-informative PCs.

*correlation is significant at .05 level.

**correlation is significant at .01 level**.**

***correlation is significant at .001 level**.**

1 Handedness is a dichotomous variable with 0 =  right-handed, and 1 =  left handed/ambidextrous.

2 Marital Status is a dichotomous variable with 0 =  married/cohabiting and 1 =  single/legal separation or divorced.

3 Life Satisfaction is an ordinal variable with 0 =  very satisfied, 1 =  quite satisfied, and 2 =  quite unsatisfied/very unsatisfied.

4 Self-rated health is an ordinal ranging from 0 (very good) to 3 (bad/very bad).

Correcting for ancestry by controlling for 1, 5, or 10 ancestry-informative PCs tends to attenuate the associations; when controlling for 1 PC the relationships between the inbreeding measure and grip strength as well as life satisfaction are no longer significant, and when controlling for 5 PCs the relationship between inbreeding and birth length is no longer significant. Accordingly, when controlling for 5 or 10 ancestry PCs, five of the 18 traits (height, household income, educational attainment, and the Physical and Social Anhedonia Scales) remain correlated significantly in the expected direction – this is more than expected by chance (p = 1.5*10^−3^). When controlling for ancestry, the correlation between the inbreeding measure and the lifetime health problems scale remained significant (in the unexpected direction).

Variables for which males and females are likely to have been subject to different selection pressures were analysed separately by sex in addition to the main analysis with sexes pooled (Table 3). For height there was a significant association with level of inbreeding for both sexes, but when correcting for ancestry the effect became nonsignificant for females. For waist-to-hip ratio no significant associations were found with inbreeding for either sex. For grip-strength, we only found a significant relationship with level of inbreeding for males if ancestry was not controlled for.

**Table 3:** Correlations between inbreeding coefficients (runs of homozygosity; ROHs) and height, waist-to-hip ratio, and grip strength, for males and females separately.

	N	ROHs	ROHs corrected for 1PC	ROHs corrected for 5 PCs	ROHs corrected for 10 PCs
Height					
Males	2539	−.094***	−.081***	−.061**	−.060**
Females	2768	−.074***	−.056**	−.020	−.015
Waist-to-hip ratio					
Males	2519	.003	−.014	.006	.001
Females	2601	.009	.006	.004	.001
Grip strength					
Males	2478	−.050*	−.016	−.025	−.024
Females	2717	−.019	.003	−.001	.002

Analyses were performed without correcting for ancestry, and with correcting for ancestry by including the first, the first 5, and the first 10 ancestry-informative PCs.

*correlation is significant at .05 level.

**correlation is significant at .01 level**.**

***correlation is significant at .001 level.

## Discussion

Based on evolutionary genetic theory, we predicted that inbreeding (autozygosity, indexed by runs of homozygosity) would be associated with traits that have been under directional selection. For between five and eight of the 18 traits that we tested (depending on stringency of ancestry control), we detected significant inbreeding effects in the predicted direction: that is, higher inbreeding was associated with the lower-fitness ends of the traits. This proportion of significant inbreeding effects is significantly more than the one or two expected by chance due to multiple testing.

Several of these traits, or similar traits, had been previously linked with inbreeding, either via estimates of consanguinity or genetic autozygosity. Shorter height had recently been linked with autozygosity [15], and we replicated that here and additionally showed that the inbreeding effect was robustly observed (i.e. both before and after controlling for ancestry) only for male height, which is noteworthy in the context of other findings that tall men but not tall women are preferred as mates [28]. Further, we made the novel finding that autozygosity is also associated with lower birth length, although the association loses significance under the more stringent control of ancestry (i.e. controlling for 5 and 10 PCs). Grip strength also showed significant association (only in males, as expected), but only if ancestry-informative PCs are not controlled for. We note again that very stringent control of ancestry runs the risk of removing true association signals as well as potentially correcting for spurious association.

Schizophrenia has previously been linked with inbreeding, both via consanguinity [e.g. 6] and autozygosity [14], and schizophrenia has been shown to occur at higher rates in isolated populations [31]. We made the novel finding that continuous measures of schizotypal features (i.e. social and physical anhedonia) are associated with autozygosity. Anhedonia has been considered a key symptom of schizophrenia. Our results are consistent with the view that clinical schizophrenia reflects the extreme of a continuous spectrum of symptoms and underlying genetic load [32].

Inbreeding has previously been associated with lower intelligence (IQ) via consanguinity studies [4], [5], consistent with expectations of inbreeding depression. It is well established that IQ is highly correlated with educational attainment, so it was expected that genotype-based estimates of inbreeding would also be negatively correlated with educational attainment. A recent result in a British sample showing an association in the opposite direction [17] – higher autozygosity with higher intelligence – was surprising. Here, in a much larger sample, we show the predicted significant negative association of autozygosity with educational attainment, which was also observed in the Netherlands [18]. However, while indexing inbreeding by autozygosity reduces the threat of some of the more severe potential confounders associated with consanguinity studies (i.e. those mating with close relatives potentially being different from those who do not), there is still potential for confounding in this result. For example, inbreeding is higher and educational opportunities lower in smaller towns/communities, and those with higher educational attainment may be more likely to have moved from their birthplace and therefore mate with a more distantly related individual [18]. Our novel finding of an inbreeding effect on income is subject to the same caveats as for education, and should be likewise interpreted with caution.

Nine of the 18 traits we tested did not show a significant association with inbreeding. This is not surprising even if true inbreeding effects were pervasive, because our sample is not large enough to afford sufficient power to detect all the existing effects if they are of the small size predicted in outbred populations [11]. Nevertheless, our null findings can be informative: for example, a previous finding of an association between autozygosity and blood pressure [33] was not replicated here, even though our study employed far higher quality (denser) genotyping and a much larger sample of participants. We should have had ample power to detect effects of the size detected in Campbell et al. [33], so our null association suggests the previous finding for blood pressure is likely to have overestimated any inbreeding effect or may reflect a false-positive association.

A perplexing result is the significant *negative* association between inbreeding and lifetime health problems. One of the most straightforward predictions of inbreeding depression is a detriment to health, whereas we find greater inbreeding is associated with fewer doctor-verified health problems, both before and after controlling for ancestry stratification. Cancer, condyloma, and fractures were the only individual health problems that were significantly associated with inbreeding (all negatively) – given that 32 health problems comprised the checklist, three significant effects could easily be due to chance. Similarly, the negative association of inbreeding with the overall health problems scale could be due to chance, but the observed pattern of results renders highly unlikely the predicted inbreeding depression effect on doctor verified health problems.

Overall, our results are generally consistent with evolutionary genetic expectations regarding inbreeding depression in humans; however, the results should be interpreted with caution because of the aforementioned alternative explanations for certain traits, and the unexpected positive association of inbreeding with a scale measuring lifetime health problems. Nevertheless, our study is the first study testing the association of a genotype-based inbreeding measure with a range of physical and psychological traits in the same large sample. It therefore represents an important point of reference for further investigation of inbreeding depression in humans, which should investigate these and other traits in populations with different genetic structure and in samples that allow competing explanations to be tested.

## Supporting Information

File S1 **Supporting Information File.** Supplementary Methods. Detailed information about the measures and the data cleaning steps per measure. Supplementary Table S1. Information on the quality control procedure of the genotype data. Supplementary Table S2. Details of the parameters used for the runs of homozygosity analysis. Supplementary Material: Descriptive Statistics. Descriptive statistics of all phenotypic measures.
(DOCX)
