# Supplementary material for: The Association of Genotype-Based Inbreeding Coefficient with a Range of Physical and Psychological Human Traits
Source: PLoS One. 2014 Jul 25;9(7):e103102. doi: 10.1371/journal.pone.0103102 (PMC4111285; doi:10.1371/journal.pone.0103102)
Supplement: File S1 — Supporting Information File. Supplementary Methods. Detailed information about the measures and the data cleaning steps per measure. Supplementary Table S1. Information on the quality control procedure of the genotype data. Supplementary Table S2. Details of the parameters used for the runs of homozygosity analysis. Supplementary Material: Descriptive Statistics. Descriptive statistics of all phenotypic measures. (DOCX) [file pone.0103102.s001.docx]

**Supplementary File S1**

Karin J.H. Verweij, Abdel Abdellaoui, Juha Veijola, Sylvain Sebert, Markku Koiranen, Matthew C. Keller, Marjo-Riitta Järvelin, & Brendan P. Zietsch. The association of genotype-based inbreeding coefficient with a range of physical and psychological human traits

**Supporting Information Legends**

Supplementary Methods. Detailed information about the measures and the data cleaning steps per measure.

Supplementary Table S1. Information on the quality control procedure of the genotype data.

Supplementary Table S2. Details of the parameters used for the runs of homozygosity analysis.

Supplementary Material: Descriptive Statistics. Descriptive statistics of all phenotypic measures.

Supplementary Methods: Description of the measures

*Measures from the postal questionnaire:*

1. Marital Status was measured with the following questionnaire item:

*What is your marital status:*

*1. Married*

*2. Cohabiting*

*3. Single*

*4. Legal separation or divorced*

*5. Widowing*

*Marital Status* was converted into a dichotomous variable with 0=married/cohabiting and 1=single/legal separation or divorced. ‘Widowing’ was treated as missing.

2. Educational attainment was measured with the following questionnaire item:

*What is* y*our occupational education?*

*1. No occupation education*

*2. Vocational training course*

*3. Vocational school*

*4. Post-secondary education*

*5. Polytechnic education*

*6. University degree*

*7. Some other education*

*8. Education unfinished*

*Secondary education* was standardised separately by sex and analysed as a continuous variable. ‘Some other education’ and ‘education unfinished’ were treated as missing.

3. Income was measured with the following open question:

*What was the gross income of your household last year (gross income – including tax(in FIM/year))*

*Income* was standardised separately by sex and winsorised at 3 SDs from the mean.

4. Life Satisfaction was measured with the following questionnaire item:

*How do you feel about your current life situation in general?*

*1. Very satisfied*

*2. Quite satisfied*

*3. Quite unsatisfied*

*4. Very unsatisfied*

*5. Cannot say*

*Life Satisfaction* was converted into an ordinal variable with three categories, where 0=very satisfied, 1=quite satisfied, and 2=quite unsatisfied/very unsatisfied. ‘Cannot say’ was treated as missing.

5. Handedness was measured with the following questionnaire item:

*Are you:*

*1. Right-handed*

*2. Left-handed*

*3. Capable of using both hands equally well*

*Handedness* was converted into a dichotomous variable, where 0= right-handed, and 1=left handed/ambidextrous.

6. Self-rated health was measured with the following questionnaire item:

*What is your own estimate about your health right now?*

*1. Very good*

*2. Good*

*3. Moderate*

*4. Bad*

*5. Very bad*

*Self-rated health* was converted into an ordinal variable with 4 categories, where 0=very good, 1=good, 2=moderate, and 3=bad/very bad. Then the variable was standardised separately for males and females.

7. Lifetime health problems was measured with the following question:

*Have you ever had any of the following symptoms, sicknesses, or injuries verified or treated* *by a doctor?*

Response options were: 1) no, 2) yes.

*1. Elevated blood pressure, hypertension*

*2. Congenital heart disease*

*3. Cardiac insufficiency*

*4. Chest pain up on strain (angina pectoris)*

*5. Diabetes*

*6. Thyroiditis*

*7. Gastric or duodenal ulcer*

*8. Gallstones, gallbladder inflammation*

*9. Long-lasting of urinary tract infection mephitis*

*10. Ovaritis (women)*

*11. Prostatis (men)*

*12. Chlamydia infection*

*13. Condyloma*

*14. Herpes in genitals*

*15. Other infection in genitals*

*16. Aural infection*

*17. Ophthalmologic defect*

*18. Epilepsy*

*19. Migraine*

*20. Other neurological disease*

*21. Rheumatoid arthritis*

*22. Other arthritic condition*

*23. Degenerative or other back condition*

*24. Cancer*

*25. Hernia*

*26. Anaemia (low haemoglobin)*

*27. Mental illness, psychosis*

*28. Depression*

*29. Other mental health problem*

*30. Problems with alcohol*

*31. Other intoxicant problem*

*32. Fractures*

*33. Defect of occlusion requiring orthodontics treatment*

The *Lifetime health problems* variable was created by adding up the item responses. The sum scores were standardised separately by sex and outliers were winsorised at 3 SDs from the mean.

*Measures from the clinical examination:*

8 & 9. Systolic and diastolic blood pressure (in Hg) were measured at a clinical examination. Both measures were taken twice.

*Systolic and diastolic blood pressure* were analysed as continuous measures. Extreme outliers were removed (systolic blood pressure <= 50 mm Hg and >=230mm Hg, and diastolic blood pressure <=35 mm Hg and >=140 mm Hg), and individuals’ values were treated as missing if there was a large difference between the two systolic or the two diastolic measures (>=25 mm Hg). Subsequently, the two measures were averaged, standardised separately by sex, and outliers were winsorised at 3 SDs from the mean.

10. Heart rate (per 30 seconds) was measured at a clinical examination, while the participant was in a sitting position.

*Heart rate* was analysed as a continuous measure. Extreme outliers were removed (>=75 beats per 30 seconds). Subsequently, the variable was standardised separately by sex, and outliers were winsorised at 3 SDs from the mean.

11. Height (in cm with an accuracy of 0.1 cm) was measured at a clinical examination.

*Height* was analysed as a continuous measure. Extreme outliers were removed (height < 1.30 meters). The variable was standardised separately by sex and outliers were winsorised at 3 SDs from the mean.

12. BMI was derived from height and weight (in kg with an accuracy of 0.1kg) obtained at a clinical examination. BMI was calculated by dividing weight in kg by height^2^ (in meters).

*BMI* was analysed as a continuous variable. Extreme outliers for height (see above) and weight (weight <= 40kg) were removed. Subsequently, BMI was standardised separately by sex and outliers were winsorised at 3 SDs from the mean.

13. Waist-to-hip ratio was derived from hip and waist circumference measures (both in cm with an accuracy of 0.5 cm) obtained at a clinical examination. Waist-to-hip ratio was calculated as waist circumference divided by hip circumference.

Past research has found that a waist-to-hip ratio of 0.7 is optimal, but only 4 males (0.2%) and 47 females (1.8%) had a waist-to-hip-ratio below 0.7, rendering it not worthwhile to use a ‘difference from optimum’ score.

*Waist-to-hip ratio* was analysed as a continuous variable. The variable was standardised separately by sex and outliers were winsorised at 3 SDs from the mean.

14. Gripstrength (in kg with an accuracy of 0.1kg) was measured three times at a clinical examination.

The *Grip strength* variable comprised a composite measure of the three grip strength tests and was analysed as a continuous variable. Multivariate outliers were identified (using the Mahar test for multivariate outliers) and treated as missing. Subsequently, the three grip strength measures were averaged, standardised separately by sex, and winsorised at 3 SDs from the mean.

15. Birth-length was measured on a seven point Likert scale ranging from 1 (shorter than two standard deviations below the mean) to 7 (taller than 2 standard deviations above the mean).

*Birth-length* was analysed as a continuous variable. The variable was standardised separately by sex.

16. Physical Anhedonia Scale was measured with the Revised Physical Anhedonia Scale (PAS, see Chapman et al, 1976), a 61 item scale measuring individuals’ ability to experience physical and sensory pleasures. Items were answered with true or false. Scale scores were derived by adding up the total item scores, with high scores indicating lowered ability to experience physical and sensory pleasures. If an individual had more than 10% missing items, the variable was treated as missing data; otherwise missing items were imputed based on the individual’s responses to the other items.

The internal consistency of this measure (as determined in the current sample) is high (see Miettunen et al., 2010).

*Physical Anhedonia Scale* was analysed as a continuous variable. The variable was standardised separately by sex, and outliers were winsorised at 3 SDs from the mean.

17. Social Anhedonia Scale was measured with the Revised Social Anhedonia Scale (SAS, see Chapman et al, 1976; Eckblad et al, 1982), a 40 item scale measuring individuals’ interest in social interaction. Items were answered with true or false. Scale scores were derived by adding up the total item scores, with high scores corresponding to schizoid lack of interest in social interaction. If an individual had more than 10% missing items, the variable was treated as missing data; otherwise missing items were imputed based on the individual’s responses to the other items.

The internal consistency of this measure (as determined in the current sample) is high (see Miettunen et al., 2010).

*Social Anhedonia Scale* was analysed as a continuous variable. The variable was standardised separately by sex, and outliers were winsorised at 3 SDs from the mean.

18. Perceptual Aberration Scale was measured with the Perceptual Aberration Scale (PER, see Chapman et al, 1978), a 35 item scale measuring individuals’ perception of their own body and other objects. Items were answered with true or false. Scale scores were derived by adding up the total item scores, with high scores corresponding to a distorted perception of own body and other objects. If an individual had more than 10% missing items, the variable was treated as missing data; otherwise missing items were imputed based on the individual’s responses to the other items.

The internal consistency of this measure (as determined in the current sample) is high (see Miettunen et al., 2010).

*Perceptual Aberration Scale* was analysed as a continuous variable. The variable was standardised separately by sex, and outliers were winsorised at 3 SDs from the mean.

Supplementary Table S1. Genotype quality control information. Table includes initial number of SNPs and individuals, individuals not passing quality control (QC), SNPs not passing QC for Hardy-Weinberg equilibrium (HWE), minor allele frequency (MAF) and SNP call rate, number of SNPs and individuals after QC, and SNPs removed and retained after light pruning.

| **Initial # of individuals** | **Initial # of autosomal SNPs** | **Individuals removed** | **HWE**  **p<10^-3^** | **MAF <.05** | **SNP call rate <95%** | **Individuals after QC** | **SNPs after QC** | **SNPs removed after light pruning** | **SNPs retained after light pruning** |
| --- | --- | --- | --- | --- | --- | --- | --- | --- | --- |
| 5,546 | 355,635 | 178 | 6,494 | 40,293 | 2,331 | 5,368 | 309,315 | 124,406 | 184,909 |

Light LD pruning: Removal of SNPs using PLINK with the following parameters: window size in SNPs = 50, number of SNPs to shift the window at each step = 5, VIF > 10 (r^2^>0.9)

Supplementary Table S2. Parameters used for the Runs-of-Homozygosity analysis in PLINK, based on recommendations from Howrigan et al. (2011).

| **ROH analysis, function parameters** | **PLINK command** | **Value used** |
| --- | --- | --- |
| - SNP threshold to call a ROH | --homozyg-snp | 65 (after light pruning of SNPs) |
| - Sliding window size in SNPs | --homozyg-window-snp | 65 (after light pruning of SNPs) |
| - Heterozygote allowance | --homozyg-window-het | 0 |
| - Missing SNP allowance | --homozyg-window-missing | 3 |
| - Window threshold to call a ROH | --homozyg-window-threshold | 0.05% of SNP threshold |
| - Sliding window size in kb | --homozyg-window-kb | 0 (unused) |
| - Kb threshold to call a ROH | --homozyg-kb | 0 (unused) |
| - Minimum SNP density to call a ROH | --homozyg-density (kb) | 5,000 (set high to ignore) |
| - Maximum gap before splitting ROH | --homozyg-gap (kb) | 5,000 (set high to ignore) |

Supplementary Material: Descriptive Statistics

*Continuous variables* (after excluding outliers, before standardising, winsorising)

| Phenotype | Males | | Females | |
| --- | --- | --- | --- | --- |
|  | N | M (SD) | N | M (SD) |
| Height | 2539 | 178.21 (6.40) | 2768 | 164.77 (6.04) |
| BMI | 2538 | 25.21 (3.59) | 2751 | 24.19 (4.70) |
| Waist-to-hip ratio | 2519 | 0.91 (0.06) | 2601 | 0.81 (0.08) |
| Diastolic blood pressure | 2527 | 80.39 (11.33) | 2752 | 74.63 (10.82) |
| Systolic blood pressure | 2530 | 130.28 (12.69) | 2759 | 119.84 (12.24) |
| Heart rate | 2525 | 33.76 (5.27) | 2762 | 35.81 (5.23) |
| Grip strength | 2478 | 47.19 (8.73) | 2717 | 26.68 (6.03) |
| Household income | 2316 | 168,483 (86,545) | 2472 | 177,451 (171,302) |
| Lifetime health problems | 2489 | 1.79 (1.51) | 2692 | 2.29 (1.84) |
| Physical Anhedonia Scale | 2032 | 17.90 (7.38) | 2500 | 12.66 (5.89) |
| Social Anhedonia Scale | 2030 | 11.07 (5.96) | 2500 | 8.15 (4.78) |
| Perceptual Aberration Scale | 2030 | 2.10 (3.20) | 2500 | 2.60 (3.36) |

*Ordinal/dichotomous variables:*

Marital status:

|  |  | Males (N=2538) | Females (N=2766) |
| --- | --- | --- | --- |
| 1 | Married | 1113 (43.9%) | 1457 (52.7%) |
| 2 | Cohabiting | 642 (25.3%) | 650 (23.5%) |
| 3 | Single | 696 (27.4%) | 515 (18.6%) |
| 4 | Divorced/separated | 87 (3.4%) | 144 (5.2%) |
| 5 | Widow* | 0 | 3 |
| **0** | **In a relationship** | 1755 (69.1%) | 2107 (76.2%) |
| **1** | **Single** | 783 (30.9%) | 659 (23.8%) |

**Not included in analyses*

Educational attainment:

|  |  | Males (N=2299) | Females (N=2310) |
| --- | --- | --- | --- |
| 1 | No occupation education | 182 (7.9%) | 165 (7.1%) |
| 2 | Vocational training course | 140 (6.1%) | 128 (5.5%) |
| 3 | Vocational school | 1061 (46.2%) | 552 (23.9%) |
| 4 | Post-secondary education | 611 (26.6%) | 1148 (49.7%) |
| 5 | Polytechnic education | 56 (2.4%) | 31 (1.3%) |
| 6 | University degree | 249 (10.8%) | 286 (12.4%) |
| 7 | Some other education* | 133 | 317 |
| 8 | Education unfinished* | 93 | 133 |

**Not included in analyses*

Handedness:

|  |  | Males (N=2541) | Females (N=2779) |
| --- | --- | --- | --- |
| 1 | Right-handed | 2296 (90.4%) | 2591 (93.2%) |
| 2 | Left-handed | 204 (8.0%) | 169 (6.1%) |
| 3 | Ambidextrous | 41 (1.6%) | 19 (0.7%) |
| **0** | **Right-handed** | 2296 (90.4%) | 2591 (93.2%) |
| **1** | **Non right-handed** | 245 (9.6%) | 188 (6.8%) |

Life satisfaction:

|  |  | Males (N=2479) | Females (N=2723) |
| --- | --- | --- | --- |
| 1 | Very satisfied | 499 (20.1%) | 627 (23.0%) |
| 2 | Quite satisfied | 1702 (68.7%) | 1901 (69.8%) |
| 3 | Quite unsatisfied | 243 (9.8%) | 169 (6.2%) |
| 4 | Very unsatisfied | 35 (1.4%) | 26 (1.0%) |
| 5 | Cannot say* | 46 | 39 |
| **0** | **Very satisfied** | 499 (20.1%) | 627 (23.0%) |
| **1** | **Quite satisfied** | 1702 (68.7%) | 1901 (69.8%) |
| **2** | **Quite/very unsatisfied** | 278 (11.2%) | 195 (7.2%) |

*Not included in analyses*

Self-rated health:

|  |  | Males (N=2535) | Females (N=2767) |
| --- | --- | --- | --- |
| 1 | Very good | 358 (14.1%) | 340 (12.3%) |
| 2 | Good | 1331 (52.5%) | 1486 (53.7%) |
| 3 | Moderate | 765 (30.2%) | 846 (30.6%) |
| 4 | Bad | 70 (2.8%) | 89 (3.2%) |
| 5 | Very bad | 11 (0.4%) | 6 (0.2%) |
| **0** | **Very good** | 358 (14.1%) | 340 (12.3%) |
| **1** | **Good** | 1331 (52.5%) | 1486 (53.7%) |
| **2** | **Moderate** | 765 (30.2%) | 846 (30.6%) |
| **3** | **Bad/very bad** | 81 (3.2%) | 95 (3.4%) |

Birth length:

|  |  | Males (N=2453) | Females (N=2652) |
| --- | --- | --- | --- |
| 0 | < 2 SDs below the mean | 28 (1.1%) | 41 (1.5%) |
| 1 | 2 SDs to 10^th^ percentile | 400 (16.3%) | 322 (12.1%) |
| 2 | 10^th^ to 25^th^ percentile | 471 (19.2%) | 505 (19.0%) |
| 3 | 25^th^-75^th^ percentile | 1144 (46.6%) | 1314 (49.5%) |
| 4 | 75^th^ to 90^th^ percentile | 235 (9.6%) | 329 (12.4%) |
| 5 | 90^th^ percentile to 2 SDs above the mean | 125 (5.1%) | 102 (3.8%) |
| 6 | > 2 SDs above the mean | 50 (2.0%) | 39 (1.5%) |

SD=Standard deviation

**References Supplementary Material**

Chapman, L.J., Chapman, J.P., & Raulin, M.L. (1976). Scales for physical and social anhedonia. *Journal of Abnormal Psychology, 85(4)*:374–382.

Chapman, L.J., Chapman, J.P., & Raulin, M.L. (1978). Body-image aberration in schizophrenia. *Journal of Abnormal Psychology, 87(4)*:399–407.

Eckblad, M., Chapman, L.J., Chapman, J.P., & Mishlove, M (1982). *The Revised Social Anhedonia Scale.* Madison, Wis, USA: University of Wisconsin.

Miettunen, J., Veijola, J., Freimer, N., Lichtermann, D., Peltonen, L., Paunio, T., Isohanni, M., Joukamaa, M., & Ekelund, J. (2010). Data on schizotypy and affective scales are gender and education dependent—study in the Northern Finland 1966 Birth Cohort. *Psychiatry Research, 178(2)*:408–41
